# LungHist700: A dataset of histological images for deep learning in pulmonary pathology

**DOI:** 10.1038/s41597-024-03944-3

**Published:** 2024-10-05

**Authors:** Jorge Diosdado, Pere Gilabert, Santi Seguí, Henar Borrego

**Affiliations:** 1 Dept. de Matemàtiques i Informàtica, Universitat de Barcelona, Barcelona, Spain; 2 Hospital Clínico Universitario de Valladolid, Valladolid, Spain

**Keywords:** Non-small-cell lung cancer, Pathology

## Abstract

Accurate detection and classification of lung malignancies are crucial for early diagnosis, treatment planning, and patient prognosis. Conventional histopathological analysis is time-consuming, limiting its clinical applicability. To address this, we present a dataset of 691 high-resolution (1200 × 1600 pixels) histopathological lung images, covering adenocarcinomas, squamous cell carcinomas, and normal tissues from 45 patients. These images are subdivided into three differentiation levels for both pathological types: well, moderately, and poorly differentiated, resulting in seven classes for classification. The dataset includes images at 20x and 40x magnification, reflecting real clinical diversity. We evaluated image classification using deep neural network and multiple instance learning approaches. Each method was used to classify images at 20x and 40x magnification into three superclasses. We achieved accuracies between 81% and 92%, depending on the method and resolution, demonstrating the dataset’s utility.

## Background & Summary

Cancer is the second leading cause of death globally. In 2022, more than 20 million new cancer cases were reported, and approximately 9.7 million people succumbed to the disease worldwide. Lung cancer, with more than 2.5 million new cases diagnosed^1^, was the most lethal, accounting for 1.8 million deaths. This staggering figure represents a fifth of all cancer deaths globally, significantly more than the second deadliest cancer, colon and rectum cancer, which caused almost 904,000 deaths in the same year, 2022^2^.

The high mortality rate of lung cancer is mainly due to late detection. Early diagnosis of lung cancer is key to survival. However, by the time symptoms become apparent, the disease has often spread, resulting in a low survival rate^3^. The 5-year survival rate for early-stage lung cancer can exceed 90%, while for patients diagnosed at a late stage, it can be less than 10%0^4^. Smoking, identified as the leading risk factor by the American Cancer Society, is projected to account for 81% of lung cancer cases in 2023^5^.

Carcinomas, malignancies that develop from epithelial cells, are the most common type of malignancy in the lungs. Carcinomas located in the lungs that originate there are referred to as primary lung carcinomas, distinguishing them from those that have spread to the lungs via metastasis. Primary lung carcinomas can be divided into two major histopathological types: small cell carcinoma and non-small cell carcinoma, with non-small cell carcinomas being the most frequent^6^.

Non-small cell carcinoma can be classified into two main subtypes: adenocarcinomas and squamous cell carcinomas.

- **Adenocarcinomas**: These tumors exhibit microscopic glandular-related tissue cytology, tissue architecture, and/or gland-related products.
- **Squamous Cell Carcinomas**: These tumors are characterized by observable traits of squamous differentiation, such as intercellular bridges, keratinization, and the formation of squamous pearls^6^.

Additionally, there are other less common types of non-small cell carcinoma, such as large cell carcinoma, adenosquamous carcinoma, and sarcomatoid carcinoma, each with its own unique histological features and clinical behaviors that may influence treatment strategies and prognosis.

Distinguishing the histological types of lung carcinomas is crucial in the era of personalized medicine, as each tumor type can be associated with different genetic alterations within the tumor itself. These genetic changes, in turn, are related to targeted therapies aimed at those specific mutations, improving the medium and long-term prognosis^7^.

Histopathological images, microscopic images of tissue samples, play a crucial role in medical diagnosis and research. They offer valuable insights into the appearance and structure of cells and tissues, enabling pathologists to accurately identify and classify diseases^8^. However, manual analysis of these images is time-consuming and prone to human error^9^. Therefore, histopathological image datasets, collections of labeled histopathological images, are essential for developing and training image analysis algorithms. These datasets provide researchers with a large and diverse set of images, facilitating the creation of artificial intelligence (AI) models that can accurately classify and diagnose diseases, thereby assisting human experts in their tasks.

The field of AI is expanding rapidly, with new applications emerging daily, particularly in the medical sector^10^. One promising application is in diagnostics, where AI can enhance both diagnostic accuracy and efficiency. AI can improve the early detection and diagnosis of lung cancer, potentially leading to better patient outcomes^11^.

To develop AI algorithms using lung histopathology images, several popular datasets are frequently utilized. Three of the most important ones are TCGA-LUAD^12^ for adenocarcinomas, TCGA-LUSC^13^ for squamous cell carcinoma, and LC25000^14^ which also includes slides from benign patients. The TCGA-LUAD and TCGA-LUSC datasets contain whole slide images (WSI) of lungs, specifically 541 slides from 478 LUAD patients and 512 slides from 478 LUSC patients.

The LC25000 dataset consists of 750 images of size 768 $\times$ 768, classified into three different categories: lung benign, lung adenocarcinoma, and lung squamous cell carcinoma, with 250 unique images in each category. Additionally, the dataset contains 500 images of the colon. All these images were then artificially augmented to create a dataset of 25,000 images. However, the absence of traceability from the original images to the augmented ones poses a challenge in accurately dividing the dataset into training, validation, and test sets. This lack of traceability can lead to potential data leakage during the training and validation stages, undermining the validity of technical conclusions drawn from studies using this dataset^15–19^.

This paper introduces a novel dataset, LungHist700, comprising 691 images of size 1200 $\times$ 1600 pixels from both normal lung tissue and primary lung carcinomas. The carcinomas are categorized into two types: adenocarcinomas and squamous cell carcinomas. Each of these types is further subclassified based on the degree of carcinoma differentiation into three levels: well differentiated, moderately differentiated, and poorly differentiated.

## Methods

Data was collected from 45 patients at Hospital Clínico de Valladolid in 2023 as part of a regular diagnostic process. The dataset consists of images of hematoxylin and eosin-stained samples extracted from pathology glass slides using a Leica DM 2000 microscope and a Leica ICC50 W microscope camera at two distinct magnifications: 20x and 40x. The field of view was meticulously selected by a pathologist to encompass representative tissue of the category. In most cases, this tissue is discernible in all four quadrants of the image.

All individuals included in the study were surgical patients, so all images are from patients with malignancies. Images classified as showing normal lung depict areas where the tumor has not spread.

For each patient, two concurrent evaluations were conducted to determine the type of tumor (adenocarcinoma or squamous cell carcinoma) and the level of differentiation (well differentiated, moderately differentiated, or poorly differentiated). The first evaluation was a morphological analysis of the tissue based on the hematoxylin and eosin-stained samples, which determined the classification of well and moderately differentiated samples. The second evaluation involved immunohistochemical tests of the tissue to determine the type of tumor (adenocarcinoma or squamous). These tests, combined with contextual information, contributed to the accurate classification of poorly differentiated categories. The tests performed were TTF1, CK7, Napsin A, P40, and CK5/6. Using the results from all the tests, a specialist pathologist classified the images into the seven classes of the dataset.

For adenocarcinomas, the differentiation grading system recommended by the College of American Pathologists^20,21^ was employed. According to their guidelines, there are three differentiation levels:

1. Well-differentiated: Tumors primarily exhibiting a lepidic pattern, with no high-grade components or less than 20% high-grade features (such as solid, micropapillary, or complex glandular patterns).
2. Moderately differentiated adenocarcinoma: Tumors mainly showing acinar or papillary patterns, with less than 20% high-grade features.
3. Poorly differentiated adenocarcinoma: Tumors that have 20% or more high-grade features.

Pulmonary squamous cell carcinoma has also traditionally been divided into well differentiated, moderately differentiated, and poorly differentiated, similar to squamous cell carcinomas of other organ systems. The degree of differentiation is generally dependent on a combination of features, such as the presence or absence of keratinization and intercellular bridges, as well as cellular pleomorphism and mitotic activity^22^. Following these guidelines, squamous cell carcinoma has been divided into the following three categories:

1. Well differentiated: These tumors exhibit keratinization, such as keratin pearls and intercellular bridges. They typically grow in sheets or nests, with polygonal cells that have round to oval nuclei, vesicular features, and eosinophilic cytoplasm. Additionally, mitotic figures and focal areas of hemorrhage or necrosis may be present.
2. Moderately differentiated: These tumors show increased cytologic atypia and mitotic activity. Although keratinization and intercellular bridges are still present, they are less prominent compared to well-differentiated tumors. Moreover, areas of hemorrhage or necrosis are more common.
3. Poorly differentiated: These tumors grow in sheets and are often unrecognizable as squamous type without immunohistochemistry. They display significant cellular pleomorphism, high mitotic activity, and extensive areas of necrosis.

**Fig. 1:**
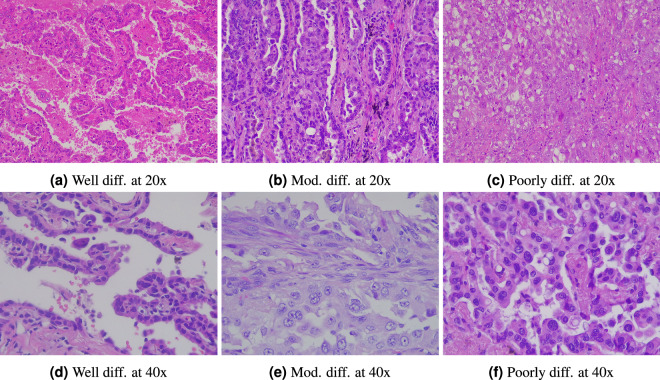
Images displaying adenocarcinoma at varying levels of differentiation and resolution.

**Fig. 2:**
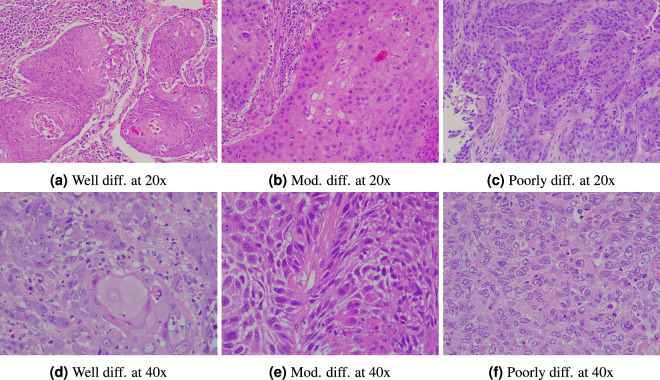
Images displaying squamous cell carcinoma at varying levels of differentiation and resolution.

**Fig. 3:**
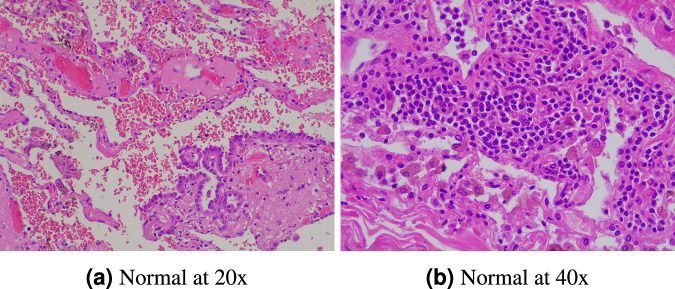
Normal lung images at different resolution.

Figure 1 shows adenocarcinoma samples, Fig. 2 displays squamous cell carcinoma samples at varying levels of differentiation and resolution. Figure 3 presents images of normal lung tissue at two different resolution.

### Ethics approval

The study was conducted according to the guidelines of the Declaration of Helsinki and approved by the Ethical Committee of the Hospital Clínico Universitario de Valladolid (CEIm Área de Salud Valladolid Este) under project PI 23–3167. The committee waived participant consent given data anonymization and approved open publication of the data.

## Data Records

The dataset is available at figshare^23^. It consists of 691 images from 45 patients, with each image having a resolution of 1200 × 1600 pixels and stored in *.jpg* format. These images are captured at either 20x or 40x magnification levels and are categorized into seven classes (see Table 1). An accompanying*.csv* file links each image to the associated patient ID. All patients have been anonymized, and the file includes an identifier to match images from the same patient.

**Table 1:** The dataset comprises three classes: adenocarcinoma (aca), squamous cell carcinoma (scc), and normal (nor).

Description	Id.	20x	40x	Subclass total	Superclass total
Well differentiated adenocarcinoma	aca_bd	57	46	**103**	**280**
Moderately differentiated adenocarcinoma	aca_md	44	46	**90**	
Poorly differentiated adenocarcinoma	aca_pd	45	42	**87**	
Normal lung	nor	85	66	**151**	**151**
Well differentiated squamous cell carcinoma	scc_bd	50	49	**99**	**260**
Moderately differentiated squamous cell carcinoma	scc_md	30	36	**66**	
Poorly differentiated squamous cell carcinoma	scc_pd	48	47	**95**	
**Total**		**359**	**332**	**691**	**691**

Images showing malignant tissue are further categorized based on their differentiation level.

## Technical Validation

In this section, we present two baseline methods for classifying the dataset into the three major superclasses. First, a classic approach was employed where images were resized, and a deep neural network (DNN) was trained. The second method involves a multiple instance learning (MIL) strategy, where patches of the images were extracted, and the same DNN was used to obtain multiple embeddings, one for each patch. An attention^24^ layer was then applied to relate and aggregate these embeddings for image classification.

All the experiments used the same training configuration: the networks were implemented using Keras and executed on an NVIDIA RTX 3090 with CUDA 11.0. The DNN model used in both methods was a ResNet50 network pretrained on ImageNet. The Adam optimizer was employed with an initial learning rate of 1e-5, which was reduced by a factor of 0.1 if the model began to overfit. Categorical cross-entropy was used as the loss function in both experiments. The Albumentations library^25^ was utilized to generate augmentations on the fly during training.

Images were classified into their superclasses: “aca” (adenocarcinoma), “scc” (squamous cell carcinoma), and “nor” (normal). The data was divided into three sets: 80% for training, 10% for validation, and the remaining 10% for testing. A patient-wise strategy was employed, ensuring that images from the same patient were placed in the same set to ensure fair evaluation and prevent data leakage.

### DNN Baseline

**Fig. 4:**
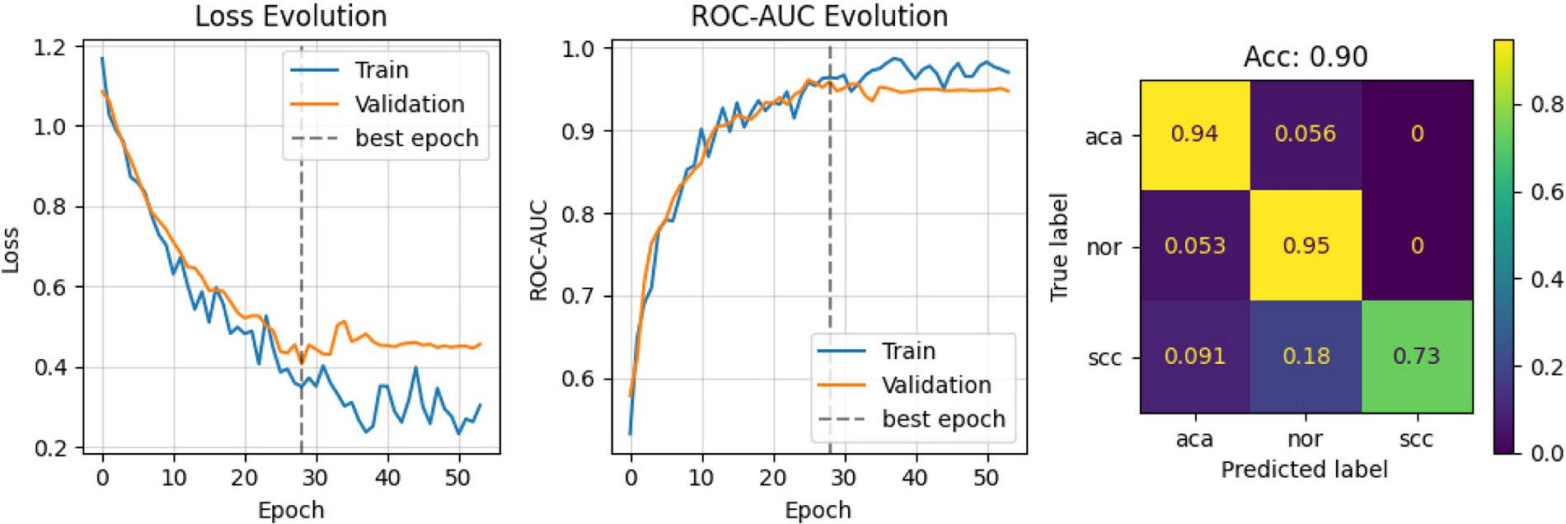
Classification results of the proposed baseline for 20x resolution. Early stopping was triggered at epoch 28, based on the validation set. After that, the best weights were loaded. The confusion matrix shows the correctly classified percentage of samples and the classification errors on the test set. The results are normalized by rows (True label).

To train the ResNet50 model, images were resized to 300 $\times$ 400 pixels to better fit this architecture. The published dataset, however, contains images at their original resolution (1200 $\times$ 1600 pixels). Figure 4 illustrates the learning curves on the training and validation splits, as well as the classification confusion matrix of the experiment on the test set for the 20x resolution. The model achieved an accuracy of 90%, a ROC-AUC of 98%, a precision of 92%, and a recall of 87%.

The experiment was then repeated with the same configuration but using images at 40x resolution. The model achieved an accuracy of 82%, a ROC-AUC of 94%, a precision of 82%, and a recall of 84%.

**Fig. 5:**
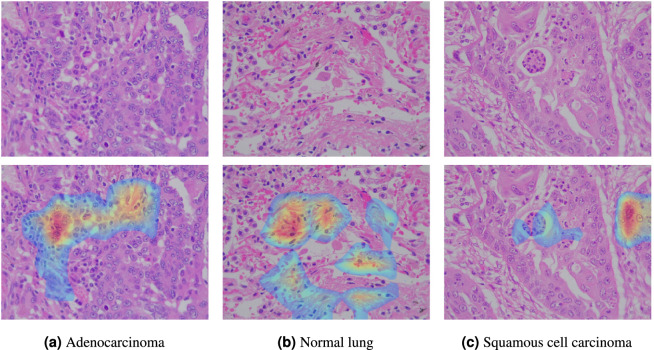
Masks generated by the Grad-CAM algorithm on some test images.

To assess the validity and explainability of the results, we used Grad-CAM^26^ on the last convolutional layer of the ResNet50 model. The threshold was set to 0.25 to visualize the Grad-CAM activations. Figure 5 shows the explanation masks generated by the algorithm on some test images, each representing a distinct histopathological class: adenocarcinoma, normal tissue, and squamous cell carcinoma. The masks illustrate how the model highlights specific areas relevant to image classification. The results were cross-checked with the medical team to validate the model’s output.

### MIL Baseline

**Fig. 6:**
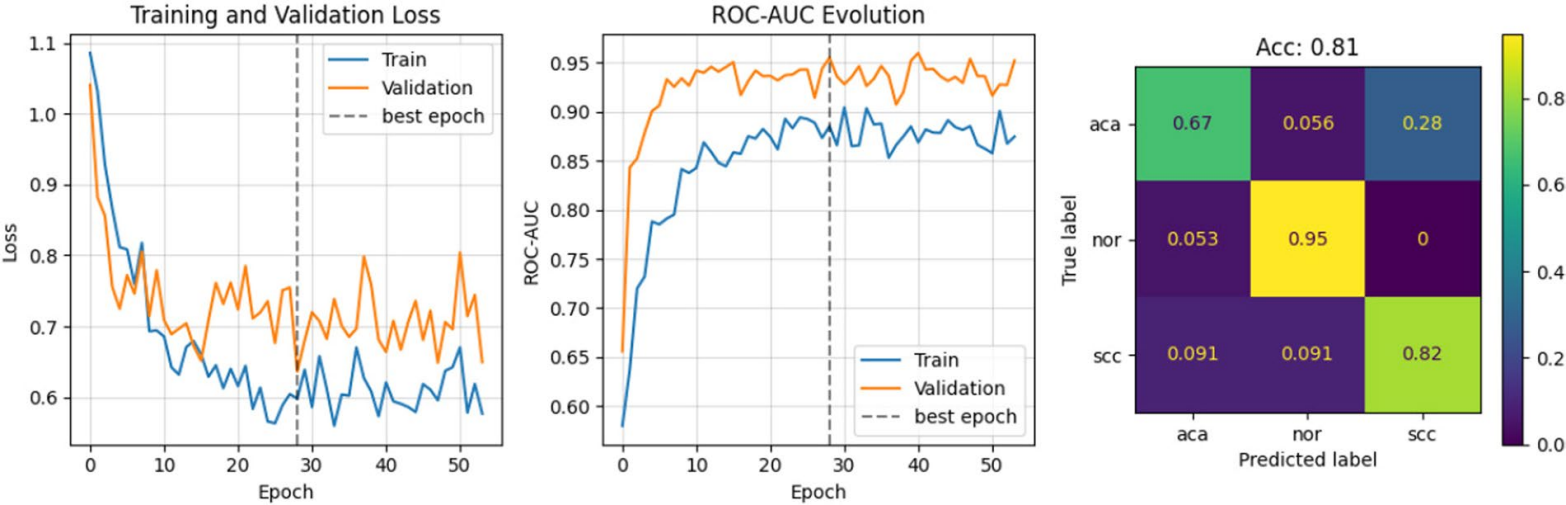
Classification performance of the MIL algorithm (ResNet50 + Multi-Head Attention layer) for 20x resolution. Early stopping was triggered at epoch 28.

A second strategy based on ResNet50 was also tested. We trained a MIL algorithm that consisted of a ResNet50 followed by a Multi-Head Attention layer. During training, we extracted 20 random patches of size 224 $\times$ 224 and used the ResNet architecture to obtain embeddings for each patch. An attention layer with four heads was then applied, followed by average pooling to obtain a single embedding for classification. All the training parameters remained the same, though the batch size was reduced to three to fit within the GPU’s memory constraints. The results of the MIL algorithm for images at 20x resolution are shown in Fig. 6. This baseline model achieved an accuracy of 81%, a ROC-AUC of 89%, a precision of 80%, and a recall of 81% on the test set.

## Data Availability

Code to reproduce the DNN baseline is available at https://github.com/jorgediosdado/LungHist700.
